# Effect of aerobic exercises on stuttering

**DOI:** 10.12669/pjms.324.9351

**Published:** 2016

**Authors:** Illays Khan, Irum Nawaz, Imran Amjad

**Affiliations:** 1 Dr. Illays Khan, DPT. Riphah College of Rehabilitation Sciences, Riphah International University, Islamabad, Pakistan; 2 Irum Nawaz, SLP/T. Riphah College of Rehabilitation Sciences, Riphah International University, Islamabad, Pakistan; 3 Dr. Imran Amjad, DPT. Riphah College of Rehabilitation Sciences, Riphah International University, Islamabad, Pakistan

**Keywords:** Aerobic exercises, Stuttering, Speech Therapy, Adolescent, Adult

## Abstract

**Background and Objective:**

Stuttering is one of the most common speech disorders in adolescents than adults. Stuttering results in depression, anxiety, behavioral problem, social isolation and communication problems in daily life. Our objective was to determine the effect of Aerobic Exercises (AE) on stuttering.

**Methods:**

A quasi trail was conducted at National Institute of Rehabilitation Medicine (NIRM) from January to June 2015. Thirty patients were selected and placed in three different groups Experimental Group A, (EG = 10 patients, age between 7-14 years), Experimental Group B (EG =10 patients age between 15-28 years) and control group –group C, (CG = 10 patients, age between 7-28 years). Patient who stutter were included in this study and those with any other pathology or comorbidity of speech disorders were excluded. The assessment tool used was Real-Time analysis of speech fluency scale. Participants in all the groups received speech therapy while only the EG – A and B received aerobic exercises (AE) using treadmill and stationary bicycle along with the speech therapy. Pre-interventional and post interventional assessments were analyzed using the SPSS 21 in order to determine the significance of new treatment approach and the effectiveness of physical therapy on speech disorders.

**Results:**

All the groups showed significant treatment effects but both the EG groups (Group A, Group B) showed high improvement in the severity level of stuttering as compared to control group C. The results also showed that AE treated group B had significant difference in p-value (p=0.027) as compared to control group (p<0.05) while experimental group A had no significant difference (p > 0.05) between these groups.

**Conclusion:**

The eclectic approach of aerobic exercises with the traditional speech therapy provides proximal rehabilitation of stuttering.

## INTRODUCTION

Stuttering which is also known as stammering is one of the most common speech disorder which is the disruption of speech motor behavior’s causing repetition and prolongation of speech sounds, syllables and words along with the repetition and prolongation of articulatory and phonatory actions.^1^ A large number of studies have been conducted considering stuttering as a speech motor disorder either on the enological pre-motor level related to cognition and linguistics or sometimes due to emotional or psychological processes.^2^ The impact of stuttering on the quality of life is negative, effecting the employment and other opportunities in life, relationships as well as job performance, and significant financial costs.^3^-^4^ Therefore stuttering is associated with high level of anxiety.^5^

Stuttering is found to be more among boys as compared to girls with a ratio of 2.4:1.^6^ Stuttering remains persistent and predominant in boys.^7^ Approximately 1% of the population stutters.^8^ In a survey of a UK population of about 1000 families, about 5% was point prevalence, about 1% annual incidence rate and the recovery rate was noted in 80% among which (4% to 5%) of the respondents showed high recovery rate from stuttering and only 20% remain persistent.^6^

Most of the studies reported two different ages of onset of stuttering as three to five years and an early onset at the age of 2 to 2-1/2 years was also noticed.^6^,^7^ Persistent stuttering also known persistent-developmental stuttering (PDS) usually occurs in children of two to four years of age which may sometime improves and remits spontaneously in affected children. Stuttering can also be found in adult population sometime secondary to a disease or neurological injury or pathology.^9^

There are many treatment approaches used worldwide for stuttering. Stuttering management started in early 19^th^ century.^10^ Over the time, adults and adolescents have mostly shown some adverse effects due to stuttering. In order to address these reactions. Cognitive behavior Therapy (CBT) is mostly used for management of stuttering resulting in their social anxiety due to stuttering. About 50% of the older population who stutter reported a high level of social anxiety and CBT mainly works in reduction of their social anxiety.^11^,^12^

A number of researches have supported that exercise benefits in executive control processing of cognition including selective attention, planning, organizing, multitasking, inhibition, along with working memory and these cognitive effects may be more distinct for women than men.^13^ In most of the studies, two different treatment approaches had been used to overcome stuttering in both children and elders.

This study aimed to provide a comprehensive approach which mainly had an affirmative positive effect on stuttering and the speech rate behavior of speakers. Mainly this study aimed at providing eclectic approach which was used collectively by both speech-language pathologists (SLPs) and physiotherapists in the treatment of both children and adults with stuttering. This study also aimed at promoting cognitive ability across a range of ages and in identifying possible predictors for the observed speech performance.

## METHODS

This study was conducted at National Institute of Rehabilitation and medicine (NIRM) Islamabad. The study protocol was approved from Ethical Committee of Riphah College of Rehabilitation, Islamabad. Thirty Urdu-speaking patients who stutter were selected through purposive sampling technique. All the participants of this study were between the ages of 7 to 28 years. Written informed consent was sought from all the participants. Patients were divided into three groups (2 treatment groups and 1 control group). Experimental group A (n=10, age 7-14 years), experimental group B (n=10, age 15-28 years) and control group (n=10, age 7-28 years). All the individuals who had a history of psychiatric or neurological disorders other than stuttering were excluded. All the participants of this study had not been involved in any stuttering treatment program for at least six months prior to participation in the experiment.

Stuttering severity of the participants of this study was analyzed by means of a Real Time Analysis of Speech Fluency scale by a team of four professional speech-language pathologists (SLPs) who had a minimum of five years of experience working with patients with stammering. It is a standardized reliable diagnostic assessment tool for speech fluency which is widely and most commonly used by SLPs around Pakistan for assessing the severity of dysfluency in spontaneous speech and reading in both English and Urdu. The score of the scale categorize the individuals who stutter into normal, borderline, mild, moderate, and severe, with a percentage syllable stammering score less than 2% which indicated normal speech and more than 12% indicating severe stuttering in the sample of 300 syllables.

Pre intervention assessment and post interventional assessment of stuttering was documented by calculating the percentage syllable stammer of individual participants by all four professional speech-language therapists for the inter-rater reliability. Pre intervention assessment of stuttering severity of all the participants was calculated in the sample of 300 syllables in spontaneous speech and reading in both English and Urdu. Experimental group A and experimental group B received aerobic exercises through treadmill and stationary bicycle under the supervision of a physical therapist for 30 minutes along with the speech therapy session of 45 minutes per session for 6 weeks (3 session per week) and control group C receiving speech therapy only for stuttering by a professional SLPs. Pre and post interventional assessment of all the participants in all three groups was analyzed using SPSS version 21.

## RESULTS

**Fig.1:**
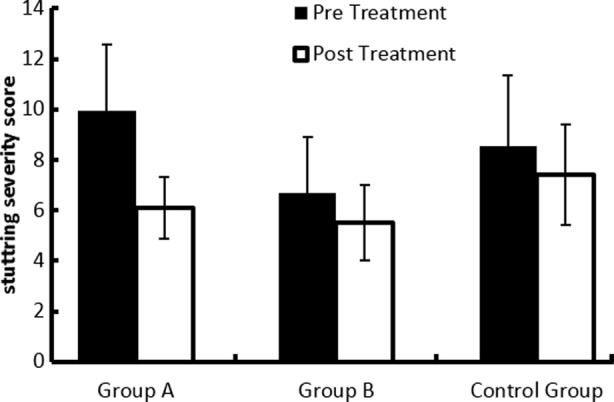
Pre and post treatment mean ± SD of stuttering severity score in speech therapy only (group C), speech therapy plus aerobic exercise group A (age between 7 to 14 year) and group B (age between 15 to 28 year).

The data collected was analyzed using statistical package for social sciences version 21. Thirty patients from NIRM were selected and placed in Experimental Group A, (EG = 10 patients, age between 7-14 years), Experimental Group B (EG =10 patients age between 15-28 years) and Control Group C, (CG = 10 patients, age between 7-28 years). The analysis of data showed the mean and standard deviation of experimental group A (receiving speech therapy and aerobic exercises), experimental group B (receiving speech therapy with aerobic exercises) and the control group C (receiving speech therapy only) indicated a significant difference (p <0.05) between their pre and post treatment values. Comparison of mean ± SD of stuttering severity scores of both the treatment groups receiving speech therapy with aerobic exercises in group A (age 7 to 14 year) and group B (15 to 28 year) showed that there was no significant difference (p > 0.05) between these groups. However the comparison of the mean ± SD of stuttering severity scores of control group C (speech therapy only) and experimental group A (speech therapy plus aerobic exercise; age between 7 to 14 year) also showed that there was no significant difference (p > 0.05) between these groups. Although the results of comparison of the mean ± SD of stuttering severity scores in control group C (speech therapy only) and experimental group B (speech therapy with aerobic exercise; age between 15 to 28 year) showed that there was a significant difference (p < 0.05) between these groups. Furthermore the mean ± SD of stuttering severity scores of pre and post treatment of all three groups (receiving speech therapy only in group C, speech therapy plus aerobic exercise in group A and group B) showed that there was a significant difference (p < 0.05) between group B and C. These results are illustrated in Fig.1.

## DISCUSSION

The study was different from previous studies in term of treatment approaches for stuttering by integrating aerobic exercises with speech therapy whereas the previous studies focused on various number of speech therapy protocols for stuttering. Speech therapy alone either in the form of comprehensive approach or other treatments have also been shown to be effective treatment for stuttering. Furthermore in this study the aerobic exercises were not a direct mode of treatment for stuttering but in collaboration with speech therapy it was helpful in improving cognitive functioning and decreasing anxiety which indirectly decreased stuttering.

The reported incidence numbers for stuttering vary, but stuttering is most common in young children (approximately 5%). Approximately 0.5% to 1% of adults stutter.^1^ These numbers indicate that most children (about 75%) recover from stuttering naturally.^14^ In this study the rate of recovery was observed more in experimental group B as compared to control group and significant difference (p < 0.05) was found.

Exercise affects humans in various ways. It is found to be a very effective treatment in children and also in improving depression as well as anxiety and self-esteem. Various processing of brain appear to be involved in protection of brain function due to physical activity. Several other molecular mediators due to exercise have a positive effect on makeup of the brain, i.e. regular exercises are found to increase the process of development of new neurons, this process is known as neurogenesis.^15^

Behavioral studies revealed that children with higher fitness level have cognitive involvement which may cause flanker task performance. The results were of significant importance because aerobic exercises cause enhancement in the cognition and increase the fitness level. Relaxation had also been reported to be directly linked with cognition.^17^-^19^

Furthermore the studies conducted on the effect of aerobic fitness among childhood indicated a direct link between one or more of the psychological processes involvement and flanker task performance. All these results raise a possibility that the changes in behavior may be due to the significant interaction between psychological and cognitive processes.^19^

Numerous studies conducted in the past have shown a positive relation between aerobic exercise and fitness on the executive functioning.^20^ Aerobic exercises cause a vital alteration in the executive functioning like learning, emotion and cognition which has a direct relation with duration of exercises and pattern of exercise. Adoption of a chronic exercise regimen causing the elevation in behavior plasticity and mood had also been observed.^21^

Further studies conducted on individuals who stutter concluded that individuals who stutter have a deficit in executive functioning which is mainly controlled by cognition. It was also revealed that planning and execution processes are distributed widely in people who stutter as compared to control non-stutter fluent speaker.^20^ In another study a significant difference was found in linguistic planning and articulation process among both fluent and non-fluent people who stutter.

Performance in motor tasks was assessed at 12 plus year of age. Furthermore motor task controls were assisted in age 12 years plus patients whose result showed poor motor control in persisted speakers compared with the recovered speakers. Severity ratings were relatively high in produced more part-word dysfluencies in persistent patients. Meta-analysis of several studies have revealed that mostly the chances of recovering naturally of the patients who stutter decreases as they reach 16 years of age and grows in teenage.^24^

Results of some other studies have also shown that the recovery rate reduces in young children as compared to adult group.^6^,^7^ The logic involved might be that due to low age the participants of the study especially age less than ten years of age was mostly unable to do aerobic exercise on treadmill and stationary bicycle which were also used in this study. Most of the children were inexperienced to both the modalities. The chance of recovery from stuttering was approximately 50% which have no relation to gender; age, in several clinical trials. The chances of recovery had direct relation to the attendance at clinic for treatment.^6^,^7^

However, the majority of studies in which comparisons were conducted showed non-significant results and the conclusion was that physical exercises have in sufficient evidence to improve cognitive function. Some research findings indicated the executive functioning is improved up to 2% due to exercise. This result was supported by another researcher who also found that 12-month exercise training program in 120 adults increased cognitive processing by 2%, which reversed 1–2 years of normal age-related atrophy. Improved cognition has a major role in behaviorally relevant improvement in spatial memory.^25^

### Limitations of the study

A small sample size due to the limited time was one of the major limitations of this study. Moreover the proper utility of sources could not be availed for measuring various aspects of stuttering, specially the cognitive effect of exercises on the sample.

## CONCLUSION

The stuttering of individuals in the age group of 15 to 28 years was better managed through eclectic approach of speech therapy with aerobic exercises than youngsters in the age group of 7 to 14 years. Moreover it was also concluded that AE are also helpful in decreasing the severity level among the individuals who stutter.

## References

[1] Max JE, Lansing AE, Koele SL, Castillo CS, Bokura H, Schachar R (2004). Attention deficit hyperactivity disorder in children and adolescents following traumatic brain injury. Dev Neuropsychol.

[2] Miller T (2015). Stuttering in the Movies: Effects on Adolescents’ Perceptions of People who Stutter.

[3] Koedoot C, Bouwmans C, Franken MC, Stolk E (2011). Quality of life in adults who stutter. J Commun Disord.

[4] Blumgart E, Tran Y, Craig A (2010). An investigation into the personal financial costs associated with stuttering. J Fluency Disord.

[5] Kraaimaat FW, Vanryckeghem M, Van Dam-Baggen R (2002). Stuttering and social anxiety. J Fluency Disord.

[6] Andrews G, Harris M (1964). The syndrome of stuttering (Clinics in Developmental Medicine).

[7] Yairi E, Ambrose NG (2005). Early childhood stuttering for clinicians by clinicians. Pro Ed.

[8] Van Riper C (1982). The nature of stuttering.

[9] Dworzynski K, Remington A, Rijsdijk F, Howell P, Plomin R (2007). Genetic etiology in cases of recovered and persistent stuttering in an unselected, longitudinal sample of young twins. Am J Speech-Language Pathol.

[10] Onslow M, Andrews C, Lincoln M (1994). A control/experimental trial of an operant treatment for early stuttering. J Speech, Language, Hearing Res.

[11] Bloodstein O (1975). Stuttering as tension and fragmentation. In Stuttering: A second symposium.

[12] Zebrowski PM, Arenas RM (2011). The “Iowa Way” revisited. J Fluency Disord.

[13] Best JR (2010). Effects of physical activity on children’s executive function: Contributions of experimental research on aerobic exercise. Develop Rev.

[14] Blomgren M (2013). Behavioral treatments for children and adults who stutter: a review. Psychol Res Behavior Management.

[15] United States. Department of Health (1996). Physical activity and health: a report of the Surgeon General.

[16] Hillman CH, Pontifex MB, Raine LB, Castelli DM, Hall EE, Kramer AF (2009). The effect of acute treadmill walking on cognitive control and academic achievement in preadolescent children. Neuroscience.

[17] Voss MW, Prakash RS, Erickson KI, Basak C, Chaddock L, Kim JS (2010). Plasticity of brain networks in a randomized intervention trial of exercise training in older adults. Front Aging Neurosci.

[18] Chaddock L, Erickson LI, Prakash RS, VanPatter M, Voss MW, Pontifex MB (2010). Basal ganglia volume is associated with aerobic fitness in preadolescent children. Dev Neurosci.

[19] Colcombe S, Kramer AF (2003). Fitness effects on the cognitive function of older adults a meta-analytic study. Psychological Sci.

[20] Swain RA, Berggren KL, Kerr AL, Patel A, Peplinski C, Sikorski AM (2012). On aerobic exercise and behavioral and neural plasticity. Brain Sci.

[21] Howell P (2007). Signs of developmental stuttering up to age eight and at 12 plus. Clin Psychol Rev.

[22] Smith PJ, Potter GG, McLaren ME, Blumenthal JA (2013). Impact of aerobic exercise on neurobehavioral outcomes. Mental Health Physical Activity.

